# Complementary Molecular and Spectroscopic Approaches for Meat Authentication: Real-Time PCR Detection of Porcine DNA and FT-NIR Analysis of Slaughter-Related Biochemical Changes

**DOI:** 10.3390/ijms27198898

**Published:** 2026-10-07

**Authors:** Diyas Myrzakozha, Elmira Karlova, Saule Meldebekova, Tolkyn Issabekova, Nurgali Rakhymbayev, Nyshanbay Konash, Aliya Yermekkyzy

**Affiliations:** 1 School of Pharmacy, Asfendiyarov Kazakh National Medical University, Tole bi 94, 050012 Almaty, Kazakhstan; myrzakozha.d@kaznmu.kz (D.M.); karlova.e@kaznmu.kz (E.K.); aliyaermek777@gmail.com (A.Y.); 2 Faculty of Medicine, Karakalpakstan Medical Institute, Dosnazarov 108, Nukus 230105, Uzbekistan; saule_meldebekova1932@mail.ru; 3 School of General Medicine, Asfendiyarov Kazakh National Medical University, Tole bi 94, 050012 Almaty, Kazakhstan; issabekova.t@kaznmu.kz; 4 Faculty of Pharmacy, JSC “ South Kazakhstan Medical Academy”, Al-Farabi Square, 1, 160000 Shymkent, Kazakhstan

**Keywords:** meat authentication, food adulteration, porcine DNA, real-time PCR, FT-NIR spectroscopy, molecular authentication, chemometrics, principal component analysis, food safety, food analysis

## Abstract

Food adulteration and species mislabeling represent important challenges for food safety, regulatory control, and consumer protection, particularly for products requiring species-specific authentication. This study investigated two complementary analytical approaches relevant to meat authentication: species-specific real-time polymerase chain reaction (real-time PCR) for the molecular detection of porcine mitochondrial deoxyribonucleic acid (DNA) and Fourier-transform near-infrared (FT-NIR) spectroscopy combined with chemometric analysis for exploratory assessment of slaughter-related compositional changes in a controlled murine proof-of-concept model. A total of 85 commercially available Halal-labeled meat products, including sausages, cutlets, minced meat, dumplings, raw meat, and seasoning products, were analyzed for the presence of porcine mitochondrial DNA. Under the applied qualitative assay conditions, porcine mitochondrial DNA was detected in 64 of 85 analyzed samples (75.3%). This finding is specific to the investigated sample set and does not distinguish intentional formulation or substitution from unintended cross-contact or other potential sources of porcine-derived DNA. In parallel, FT-NIR spectroscopy over the 10,000–4000 cm^−1^ spectral range was evaluated as a rapid, non-destructive approach for assessing compositional changes associated with blood removal in a controlled murine model. Principal component analysis of standard normal variate (SNV)-preprocessed spectra demonstrated separation between blood-retained and blood-drained tissues; the first principal component (PC1) and the second principal component (PC2) accounted for 93% and 4% of the total spectral variance, respectively. The spectral regions contributing most strongly to the observed separation were associated predominantly with O–H- and N–H- related vibrational features, consistent with variation in major tissue constituents such as water and proteins. These findings illustrate the complementary information provided by species-specific molecular detection and exploratory spectroscopic profiling. Further validation in commercially relevant livestock species and independent sample sets is required before a combined analytical workflow can be established.

## 1. Introduction

Food authenticity is an important component of food safety, quality assurance, and consumer protection. The increasing complexity of food supply chains and the extensive processing of meat products can make verification of declared animal species challenging, particularly in products containing multiple ingredients. Undeclared species substitution, accidental cross-contamination, and other forms of meat adulteration may compromise product integrity and create economic, regulatory, ethical, and dietary concerns [1,2,3]. Consequently, reliable analytical methods capable of identifying animal-derived components in both raw and processed foods are increasingly important for routine food authentication and regulatory monitoring.

Among the analytical approaches available for meat authentication, DNA-based methods provide high specificity because taxonomically informative genetic sequences can be targeted independently of many physicochemical changes occurring during food processing. Polymerase chain reaction (PCR) and real-time PCR have therefore become widely applied tools for species identification and the detection of undeclared animal components in complex food matrices [1,4]. Compared with protein-based approaches, DNA analysis can remain effective in many processed products because species-specific DNA fragments may persist after heating and manufacturing. Recent studies continue to demonstrate the applicability of DNA-based strategies to pork authentication. Yin et al. developed a pig-specific PCR–lateral flow strip assay targeting the mitochondrial *cytb* gene for rapid detection of pork and pig-derived components in meat products. The assay detected pork components at 0.01% in adulterated meat and showed no cross-reactivity with the non-target animal species evaluated [2]. Similarly, Magiati et al. developed a gold nanoparticle-based lateral flow assay for the visual detection of species-specific DNA from pork and other meat species, with porcine DNA detectable at 0.02% in binary DNA mixtures [3]. These studies illustrate the sensitivity and specificity achievable with species-specific DNA-based approaches for pork authentication. Porcine DNA is of particular analytical interest because undeclared porcine material may arise from species substitution or cross-contamination during food production and is relevant to product labeling, food integrity, and specific dietary requirements.

Molecular identification, however, primarily addresses the biological origin of a food component and does not necessarily provide information about physicochemical or compositional changes associated with processing and handling. Spectroscopic techniques can provide complementary information by characterizing the molecular and compositional properties of biological tissues. Near-infrared (NIR) spectroscopy is particularly attractive for meat analysis because it is rapid, non-destructive, requires minimal sample preparation, and provides spectral information associated predominantly with overtone and combination vibrations of O–H, C–H, and N–H bonds [5,6,7]. These spectral features reflect major constituents of meat, including water, lipids, and proteins, and can therefore be exploited for authentication and classification when combined with multivariate chemometric analysis [5,6,7,8].

Blood removal during slaughter can also modify the biochemical composition of muscle tissue. Exsanguination alters the amount and distribution of residual blood and consequently affects water- and protein-associated constituents within the tissue matrix [9]. Because water, proteins, and lipids contribute strongly to NIR absorption, such compositional changes may generate measurable spectral differences between blood-retained and blood-drained tissues [5,6]. FT-NIR spectroscopy combined with chemometric analysis may therefore provide a rapid approach for investigating slaughter-related compositional changes without requiring direct measurement of individual low-abundance compounds. Standard Normal Variate (SNV) transformation can be used to reduce scattering-related spectral variation, while Principal Component Analysis (PCA) provides an unsupervised approach for exploring multivariate spectral structure and identifying patterns contributing to sample differentiation [10,11,12].

Despite advances in both DNA-based authentication and vibrational spectroscopy, these analytical approaches are commonly applied independently. Molecular assays provide highly specific information on species identity, whereas spectroscopic techniques characterize broader biochemical and compositional differences within the food matrix. Their complementary application may therefore provide a more comprehensive analytical framework for meat authentication than either approach alone. However, studies combining species-specific molecular detection with spectroscopic assessment of slaughter-related compositional changes remain limited.

Accordingly, the present study investigated two complementary analytical approaches relevant to meat authentication, addressing distinct analytical questions rather than providing direct cross-validation of the same samples. The first objective was to apply species-specific real-time PCR to determine the occurrence of porcine mitochondrial DNA in 85 commercially available meat products labeled as Halal. The second objective was to evaluate FT-NIR spectroscopy, combined with spectral preprocessing and chemometric analysis, to explore blood-removal-associated compositional differences in a controlled murine model. The two experimental components were therefore designed to provide complementary, rather than mutually confirmatory, analytical information.

## 2. Results

### 2.1. Real-Time PCR Detection of Porcine DNA

Species-specific real-time PCR was used to assess the presence of porcine mitochondrial DNA in 85 commercially available meat and meat-containing products. The amplification curves demonstrated target-associated fluorescence signals during the PCR cycles, while the corresponding assay controls were used to confirm assay validity. The analyzed sample set comprised commercially available sausages, cutlets, minced meat, dumplings, raw meat, and seasoning products marketed as Halal at the time of sampling.

Overall, 64 of 85 samples (75.3%) yielded a positive qualitative result for porcine mitochondrial DNA, whereas 21 of 85 samples (24.7%) yielded a negative result under the applied assay conditions. Positive results were observed among several categories of processed meat and meat-containing products.

Representative results from selected samples are summarized in Table 1.

Among the representative samples shown in Table 1, porcine mitochondrial DNA was detected in beef sausage, chicken sausage, mixed minced meat, beef cutlets, lamb dumplings, beef minced meat, and seasoning mixture, whereas the representative raw beef sample tested negative.

These results indicate the detection of *Sus scrofa* mitochondrial DNA in 64 of the 85 analyzed samples under the applied qualitative assay conditions. The real-time PCR assay determines the presence or absence of the target DNA but, by itself, does not establish the amount, source, or mechanism responsible for its occurrence.

### 2.2. FT-NIR Spectral Profiles of Blood-Retained and Blood-Drained Tissues

**Figure 1:**
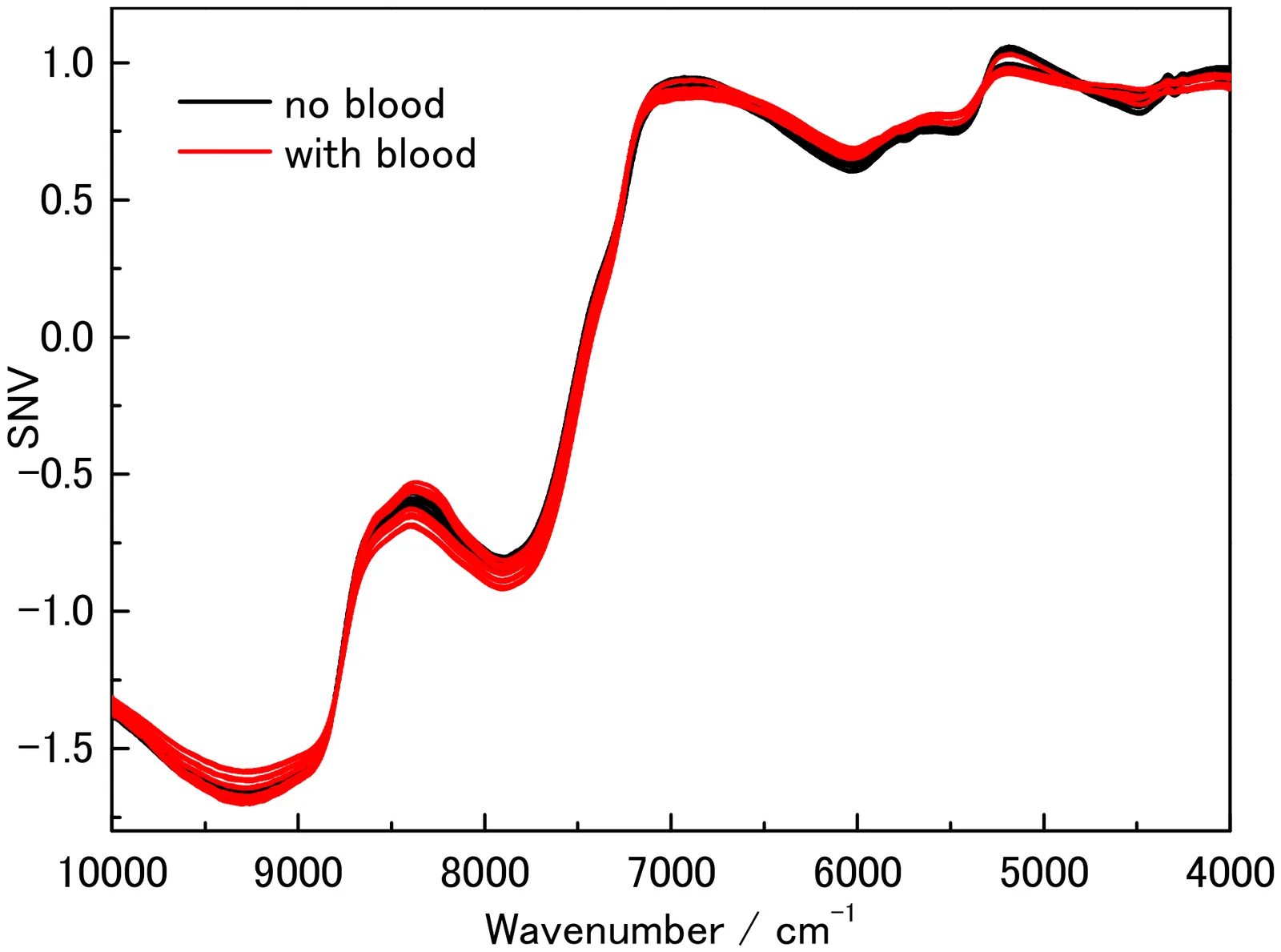
Fourier-transform near-infrared (FT-NIR) absorbance spectra of blood-retained and blood-drained murine hind-thigh tissues recorded over the spectral range of 10,000–4000 cm^−1^.

FT-NIR spectra were recorded from blood-retained and blood-drained murine hind-thigh tissues over the spectral range of 10,000–4000 cm^−1^. The raw spectral profiles of the two experimental conditions are shown in Figure 1.

The overall spectral profiles of the two groups were similar, although subtle differences were observed within several regions of the FT-NIR spectrum. A prominent spectral feature was observed around 6900 cm^−1^, with additional features observed at approximately 8330 cm^−1^ and 6550 cm^−1^.

**Figure 2:**
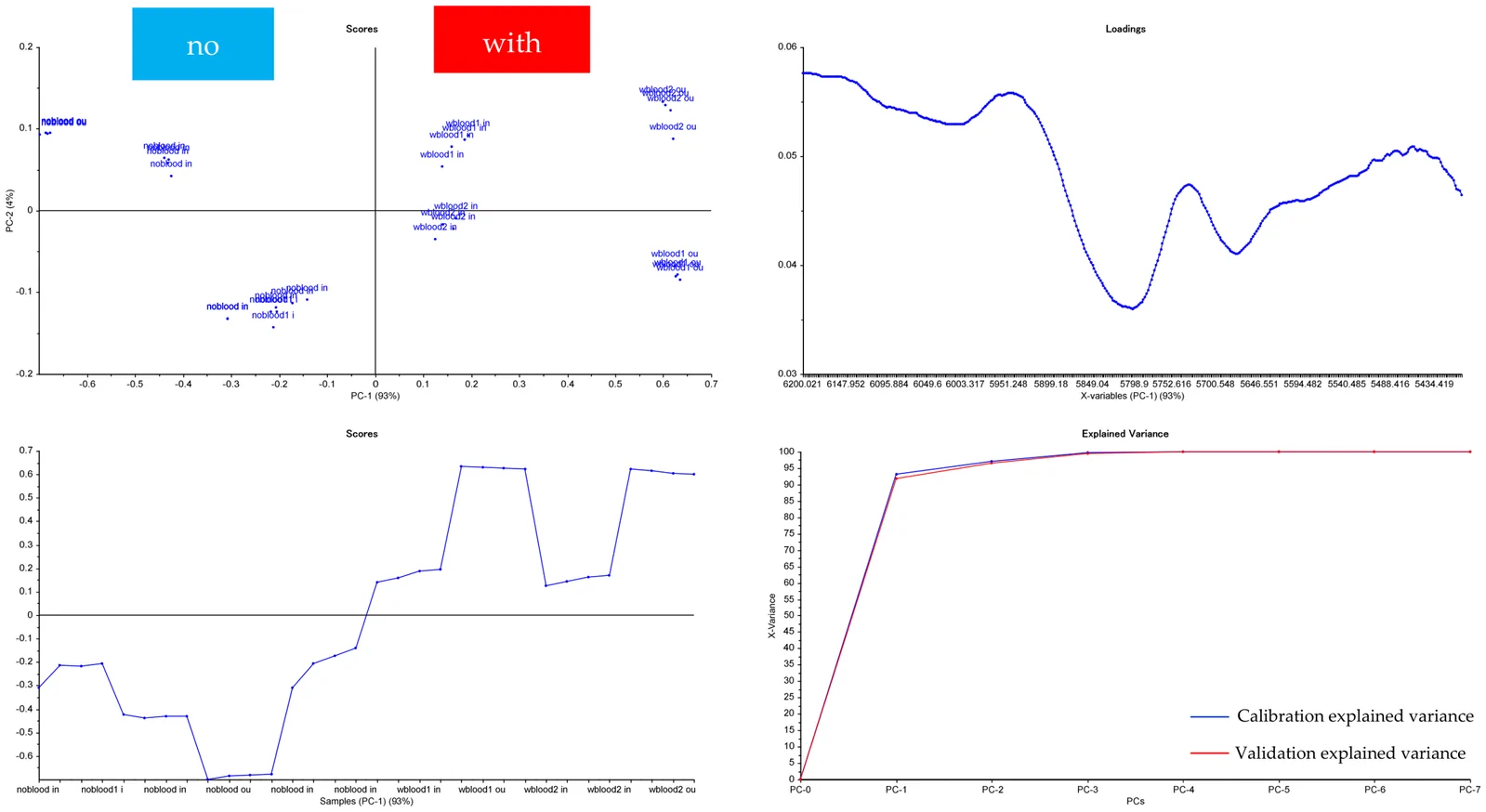
Expanded view of the standard normal variate (SNV)-preprocessed FT-NIR spectral region between 6200 and 5400 cm^−1^ for blood-retained and blood-drained murine tissue measurements. The expanded spectral interval is presented to visualize subtle group-associated differences that are difficult to distinguish in the full-range spectra shown in Figure 1. In the figure, “no” denotes the blood-drained condition and “with” denotes the blood-retained condition.

**Figure 3:**
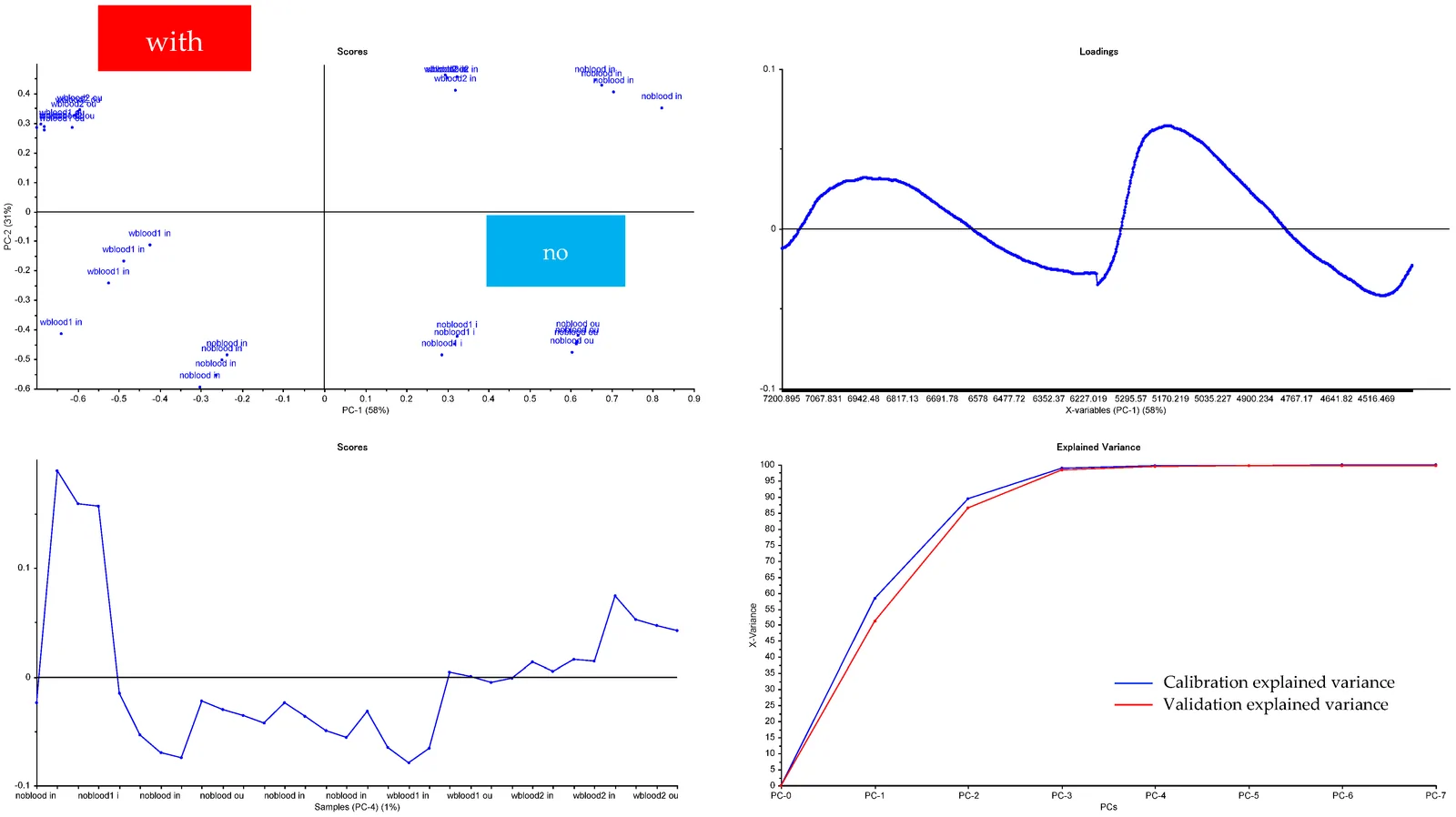
Expanded views of selected regions of the SNV-preprocessed FT-NIR spectra from blood-retained and blood-drained murine tissue measurements. The upper panel shows the 7200–6200 cm^−1^ interval and the lower panel shows the 5200–4400 cm^−1^ interval. These enlarged regions illustrate subtle spectral variation between the experimental conditions; the observed differences should not be interpreted as quantitative measurements of residual blood. In the figure, “no” denotes the blood-drained condition and “with” denotes the blood-retained condition.

To examine these differences in greater detail, selected spectral intervals were evaluated separately. Figure 2 presents the 6200–5400 cm^−1^ region, whereas Figure 3 shows selected regions within 7200–6200 cm^−1^ and 5200–4400 cm^−1^.

Following SNV preprocessing, differences between the blood-retained and blood-drained spectra became more apparent. The observed spectral variation provided the basis for subsequent multivariate analysis using PCA.

### 2.3. Principal Component Analysis of FT-NIR Spectra

**Figure 4:**
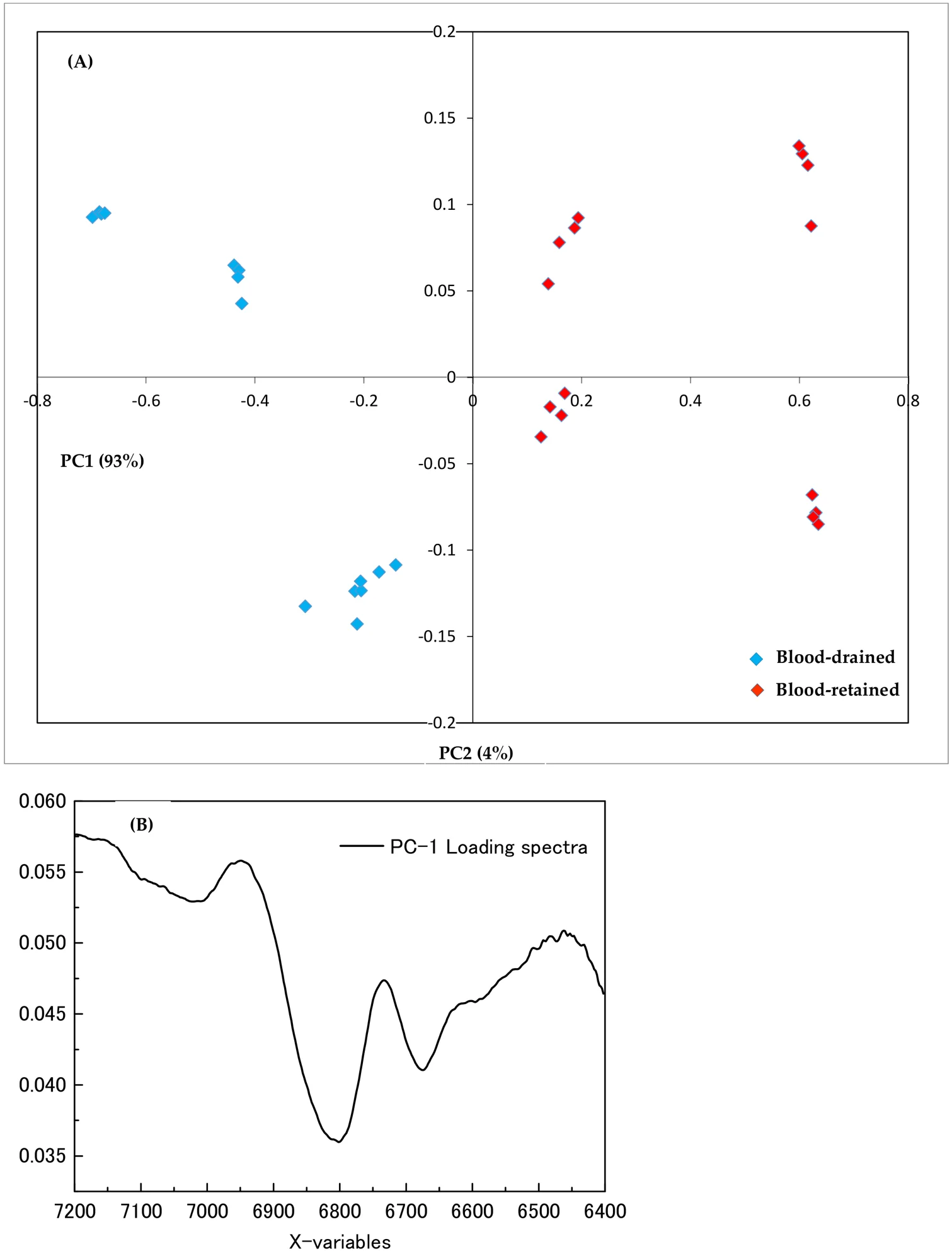
Principal component analysis (PCA) of SNV-preprocessed FT-NIR spectra from blood-retained and blood-drained murine tissue samples. (**A**) PCA scores plot showing the distribution of observations in PC1–PC2 space; the first principal component (PC1) and the second principal component (PC2) accounted for 93% and 4% of the total spectral variance, respectively. (**B**) PC1 loading profile showing the relative contribution of spectral variables to the variation represented by PC1. PCA was used for exploratory analysis; the observed group-wise separation does not represent externally validated classification performance.

PCA was applied to the preprocessed FT-NIR spectral data to evaluate multivariate variation between blood-retained and blood-drained tissue samples. The original PCA output demonstrated clustering of the samples by experimental condition (Figure 4A).

In the PCA model presented in Figure 4A, the first principal component (PC1) accounted for 93% of the spectral variance, while the second principal component (PC2) accounted for an additional 4%. Thus, the first two principal components together represented approximately 97% of the variance in this PCA model.

The PCA scores plot showed group-wise separation in PC1–PC2 space under the experimental conditions. The corresponding PC1 loading profile was examined to identify the spectral variables contributing to the observed multivariate differentiation (Figure 4B). Pronounced variation in the loading profile was observed within selected regions of the FT-NIR spectrum, indicating that specific spectral intervals contributed more strongly than others to the separation captured by PC1.

Overall, FT-NIR spectroscopy combined with spectral preprocessing and PCA revealed systematic multivariate differences between blood-retained and blood-drained murine tissue samples under the controlled conditions of this study.

## 3. Discussion

The present study investigated two complementary analytical dimensions of meat authentication: species-specific molecular identification using real-time PCR and spectroscopic characterization of compositional changes associated with blood removal. Porcine mitochondrial DNA was detected in 64 of 85 (75.3%) commercially available meat and meat-containing products analyzed in this study. In a separate controlled experiment, FT-NIR spectroscopy combined with SNV preprocessing and PCA revealed systematic multivariate differences between blood-retained and blood-drained murine tissues. Together, these findings illustrate the complementary value of highly specific DNA-based analysis and rapid spectroscopic profiling for addressing distinct aspects of meat authenticity.

### 3.1. Molecular Detection of Porcine DNA in Commercial Products

The detection of porcine DNA in 64 of 85 analyzed products demonstrates the utility of species-specific real-time PCR for identifying animal-derived material in complex commercial food matrices. DNA-based techniques are particularly useful for meat authentication because species-specific genetic targets may remain detectable in processed products in which morphological characteristics are no longer recognizable and protein-based markers may be affected by processing. Real-time PCR has consequently become an important analytical approach for detecting porcine material in both raw and processed food products [1,2,3,4].

Previous studies have demonstrated a range of DNA-based approaches for the detection and quantification of porcine material in meat products. Yin et al. developed a pig-specific PCR–lateral flow strip assay targeting the mitochondrial *cytb* gene, which detected pork components at 0.01% in experimentally adulterated meat and showed no cross-reactivity with the non-target animal species evaluated [2]. Magiati et al. reported a lateral-flow DNA assay for meat authentication in which porcine mitochondrial DNA was detectable at 0.02% in binary DNA mixtures [3]. In addition to mitochondrial targets, Chang et al. developed a real-time PCR approach based on single-copy nuclear DNA sequences for quantitative detection of pork adulteration in processed beef products [13]. Together, these studies illustrate that the analytical performance and interpretation of porcine DNA assays depend on the molecular target, assay format, sample matrix, and whether the method is designed for qualitative detection or quantitative measurement.

The present study differs from these method-development investigations because the PCR component was not designed to establish a new primer system, determine an assay-specific analytical detection limit, or quantify the proportion of porcine material. As a reference sensitivity benchmark, the manufacturer of the RapidFinder™ Pork ID Kit reports a detection limit of 0.01% (w/w) pork meat, established using standard samples consisting of mixtures of raw pork meat and other species [14]. The manufacturer further notes that the limit of detection in processed samples varies depending on sample composition and food processing conditions. Therefore, this manufacturer-reported value provides a general analytical sensitivity reference but should not be interpreted as a matrix-specific LOD for the heterogeneous commercial products examined in the present study. Instead, a commercially available species-specific detection system was applied qualitatively to a heterogeneous collection of commercial samples. Consequently, the 75.3% positivity observed in the present sample set should not be directly compared with percentage-based analytical detection limits because it represents the proportion of commercial samples yielding a qualitative positive result rather than the minimum detectable proportion of porcine material within an individual sample.

An important consideration is the interpretation of a positive porcine DNA result. Detection of *Sus scrofa* mitochondrial DNA confirms the presence of porcine-derived genetic material in the analyzed sample, but PCR alone cannot determine how that material entered the product. Potential explanations include intentional or declared formulation with porcine-derived ingredients, species substitution, ingredient-related carryover, or unintended cross-contact during processing [15]. In facilities handling multiple animal species, porcine-derived material may be transferred through shared grinders, mixers, cutting surfaces, filling equipment, utensils, storage containers, or other food-contact surfaces if cleaning and segregation procedures are insufficient. Cross-contact may also occur at different stages of the production chain, including raw-material handling and processing. Because the present study did not include environmental sampling, production-line assessment, quantitative determination of porcine material, or manufacturing traceability data, the relative contribution of these potential sources cannot be determined. Accordingly, the 75.3% positivity observed in the investigated sample set should not be interpreted as evidence that 75.3% of the products were intentionally adulterated.

This distinction is particularly important when comparing surveillance studies. The occurrence of porcine DNA can vary substantially among sample populations, product categories, geographic settings, manufacturing practices, and monitoring systems. Post-market laboratory surveillance in Thailand, for example, reported no porcine DNA in 1811 Halal-certified food samples analyzed across several food categories [16]. Such findings differ markedly from those obtained in the present sample set and demonstrate that results from individual market surveys should not be generalized to broader national or international food systems. This marked difference underscores the need to interpret the present findings specifically at the level of the investigated sample set rather than as an estimate of market-wide prevalence or as a characterization of a broader certification system.

The use of appropriate amplification controls is also critical when interpreting negative PCR findings. Food matrices may contain substances that interfere with DNA extraction or amplification, potentially producing false-negative results. Inclusion of internal and external controls therefore strengthens the analytical interpretation by demonstrating that the amplification system functioned appropriately. Recent validation studies of porcine DNA detection have similarly emphasized the importance of internal positive controls in real-time PCR-based food analysis [17].

### 3.2. FT-NIR Spectral Changes Associated with Blood Removal

The second component of the study evaluated whether blood removal produced spectroscopically detectable changes in muscle tissue. The raw FT-NIR spectra of blood-retained and blood-drained samples were broadly similar, which is expected because both groups consisted primarily of the same biological matrix. Nevertheless, subtle differences were evident in selected spectral regions, and these differences became more apparent after spectral preprocessing and multivariate analysis.

The spectral variation observed in the present study occurred within regions associated predominantly with major tissue constituents, particularly water and proteins. Because NIR bands are broad and overlapping, these features should be interpreted as part of a multicomponent spectral fingerprint rather than as compound-specific signals [5,6,7]. Particular consideration should be given to the prominent spectral contribution around 6900 cm^−1^, a region characterized by strong O–H-related absorption and sensitivity to variation in tissue water content and distribution. Given that PC1 accounted for 93% of the total spectral variance, water-related changes associated with blood removal may contribute substantially to the observed separation between the experimental conditions. The loading profiles further indicated that selected regions of the NIR spectrum contributed disproportionately to this separation. However, blood removal may concurrently alter the distribution of proteins, cellular components, and heme-containing constituents within the tissue matrix. Because tissue moisture, residual blood, and hemoglobin concentrations were not independently quantified in the present experiment, the relative contributions of these factors cannot be reliably disentangled. Accordingly, the observed spectral separation cannot be established as solely or predominantly attributable to macroscopic water loss, nor can it be specifically attributed to hemoglobin. It should instead be interpreted as an overall multivariate compositional difference associated with the blood-retained and blood-drained experimental conditions.

Previous research provides experimental support for the spectroscopic detection of residual blood. Olsen et al. investigated residual blood in Atlantic cod using chemical heme-pigment measurements together with visible and near-infrared spectroscopy. Unbled fish contained higher heme-pigment concentrations, and spectroscopic measurements showed a relationship with chemically measured residual blood; the combined VIS/NIR region produced an R^2^ of 0.83. These findings demonstrated that optical spectroscopy could capture compositional information related to residual blood in intact biological tissue [18]. Although fish muscle differs substantially from mammalian muscle and the spectral ranges differ, the study provides independent evidence supporting the general principle underlying the present proof-of-concept experiment.

At the same time, blood removal should not be interpreted as producing a simple or universally predictable change in muscle composition. Classical studies of exsanguination have shown that only part of the total blood volume is removed during slaughter and that residual blood in lean muscle is relatively low. Warriss reported that approximately 40–60% of total blood volume may be lost during exsanguination and that residual blood in lean meat is typically in the range of 2–9 mL/kg muscle [19]. Consequently, spectroscopic discrimination is likely to reflect multivariate changes in the tissue matrix rather than a direct measurement of total blood content alone.

### 3.3. Chemometric Discrimination of Blood-Retained and Blood-Drained Tissues

PCA provided a means of extracting systematic variation from the highly collinear FT-NIR dataset. In the PCA model used for the present analysis, PC1 and PC2 accounted for 93% and 4% of the spectral variance, respectively, representing approximately 97% of the variance in the first two components. The score distribution indicated separation between blood-retained and blood-drained samples, suggesting that blood removal was associated with systematic changes in the multivariate spectral fingerprint under the controlled experimental conditions.

The usefulness of chemometric analysis for meat authentication is well established. FT-NIR spectroscopy combined with multivariate analysis has previously been used to detect and quantify pork in other meat matrices. One study examining thousands of experimentally prepared meat mixtures over the 10,000–4000 cm^−1^ FT-NIR range reported that PCA captured differences among meat samples and that supervised PLS-DA and PLSR models could discriminate and quantify pork-containing mixtures [20]. More recently, NIR spectroscopy combined with chemometrics has been used to identify adulteration among pork, beef, mutton, chicken, and duck mixtures, demonstrating that subtle compositional differences can be extracted from complex meat spectra using multivariate methods [21]. Portable NIR and hyperspectral imaging have also successfully differentiated pure and adulterated alpaca meat, with PCA revealing differences between authentic and admixed samples [22].

These studies support the use of multivariate spectroscopy for food authentication but also highlight an important limitation of the present analysis. PCA is an unsupervised exploratory method; the separation observed in a PCA score plot does not by itself establish the diagnostic accuracy of a classification method. Sensitivity, specificity, prediction accuracy, and robustness cannot be inferred from visual clustering alone. No externally validated supervised classification model was established in the present study. Development of a validated screening method would require a larger independent sample set, supervised classification approaches such as PLS-DA or other appropriately validated algorithms, and external validation for objective assessment of predictive performance.

The present PCA findings should therefore be interpreted as exploratory proof-of-concept evidence that the blood-retained and blood-drained experimental conditions are associated with systematic differences in FT-NIR spectral space, rather than as validation of a diagnostic test for slaughter status or as evidence of discrimination based on a specific biochemical constituent.

### 3.4. Complementarity of Molecular and Spectroscopic Authentication

The principal conceptual contribution of the present work is the complementary use of two analytical approaches that provide fundamentally different types of information. Real-time PCR addresses species identity by detecting a species-specific molecular target, whereas FT-NIR spectroscopy characterizes the broader physicochemical composition of the tissue. The two techniques should therefore not be considered interchangeable.

PCR provides high analytical specificity but requires DNA extraction, amplification reagents, laboratory instrumentation, and appropriate contamination controls. In contrast, FT-NIR spectroscopy is rapid, reagent-free, and non-destructive once the analytical model has been established, but its spectra are inherently multivariate and require chemometric interpretation. Previous research has demonstrated the potential of NIR-based systems for rapid meat authentication and quality assessment, although translation from controlled laboratory conditions to industrial slaughterhouse environments remains challenging because of sample heterogeneity, temperature, surface properties, instrument configuration, and calibration transfer [9,20,21,22].

Combining these approaches suggests a potential tiered analytical strategy. Spectroscopic methods could ultimately provide rapid screening of large numbers of samples, while molecular assays could provide species-specific confirmation when required. The current study does not yet provide operational validation of such a workflow, but the complementary information obtained from the two analytical platforms provides a basis for further development of integrated meat-authentication systems. Importantly, because the PCR and FT-NIR components were conducted using different sample systems, the present results should not be interpreted as demonstrating cross-validation or diagnostic concordance between the two techniques.

### 3.5. Limitations and Future Research

Several limitations should be considered when interpreting the findings. First, the commercial-product component represents a defined set of 85 samples and was not designed as a statistically representative survey of all products available within a city, region, or country. Consequently, the observed frequency of porcine DNA should not be extrapolated beyond the analyzed sample set.

Second, the real-time PCR analysis was qualitative in the present study. Detection of porcine DNA demonstrates the presence of the target genetic material but does not establish its mass fraction or distinguish trace cross-contact from deliberate formulation or substitution. In addition, a matrix-specific limit of detection was not independently established in the present study. Although the manufacturer reports a detection limit of 0.01% (w/w) pork meat under defined reference conditions, this benchmark cannot be assumed to represent the matrix-specific sensitivity of the assay across the heterogeneous processed products analyzed in this study. Independent measurements of DNA purity and integrity were not available for the present analysis, although PCR amplification performance was monitored using the assay’s internal positive control. Detailed records of the initial sample mass and homogenization conditions were also not available. These methodological limitations should therefore be considered when interpreting the qualitative PCR findings. Consequently, positive results should be interpreted as qualitative detection of the target DNA under the applied assay conditions rather than as quantitative evidence of a defined proportion of porcine material. Future studies incorporating validated quantitative PCR approaches, independent DNA quality assessment, and more fully documented sample-preparation procedures could improve the analytical characterization and interpretation of positive findings.

Third, the FT-NIR experiment was conducted using a small controlled murine model. The use of laboratory animals reduced some sources of biological variability and was appropriate for an initial proof-of-concept experiment, but murine muscle cannot be assumed to reflect the spectral characteristics of commercially important livestock species. Species-specific differences in muscle biochemical composition, tissue structure, water and protein distribution, and physiological responses to blood removal may influence the resulting FT-NIR spectral profiles. Therefore, the present findings should not be directly extrapolated to commercial meat. Future validation should include cattle, sheep, poultry, and other relevant species under controlled conditions, followed by evaluation under industrial slaughter conditions.

Fourth, the present FT-NIR analysis primarily demonstrated exploratory discrimination using PCA. The absence of an external validation set precluded estimation of classification sensitivity, specificity, and predictive accuracy in the present study. Future studies should include larger sample numbers, biological replicates, independent validation sets, and supervised classification approaches such as PLS-DA or other appropriately validated algorithms. Reference measurements of residual blood or hemoglobin would also be particularly valuable because they would allow the spectroscopic signal to be quantitatively related to an independent biochemical measurement rather than being inferred from experimental group assignment alone. Tissue moisture was also not independently quantified. Consequently, the present dataset does not allow determination of the extent to which the dominant PC1 variation reflects macroscopic changes in tissue water content or distribution rather than concurrent changes in proteins, residual blood components, or other tissue constituents. Future studies combining FT-NIR measurements with independent measurements of tissue moisture, residual blood, and hemoglobin concentration would be required to resolve these contributions quantitatively. In addition, alternative spectral preprocessing strategies were not systematically compared in the present analysis. First- and second-derivative transformations can reduce baseline effects and enhance overlapping spectral features in NIR data [12]. Future studies should therefore compare derivative-based preprocessing, alone or in combination with scatter-correction approaches such as SNV, and evaluate the robustness of the resulting spectral discrimination using full-resolution spectral data and independent validation datasets.

Finally, the spectral differences observed in this study should not be attributed to direct detection of adrenaline, cortisol, or other low-abundance stress hormones. Conventional NIR spectra of meat are dominated by signals from abundant constituents such as water, proteins, and lipids. Without targeted reference measurements and dedicated calibration models, assigning the observed group separation to individual physiological stress markers would not be justified. The present results are more appropriately interpreted as reflecting overall compositional changes associated with blood retention and removal.

Further research integrating quantitative molecular assays, independent reference measurements of tissue moisture, residual blood, and hemoglobin, larger livestock datasets, and externally validated chemometric models could establish whether the complementary analytical framework explored here can be translated into practical screening and confirmation strategies for meat authentication and food safety monitoring.

## 4. Materials and Methods

### 4.1. Commercial Meat Samples and Reagents

A total of 85 commercially available meat and meat-containing products were analyzed for the presence of porcine mitochondrial DNA. The samples were obtained from retail outlets in Almaty, Kazakhstan, and included sausages, cutlets, minced meat, dumplings, raw meat, and seasoning products. The products were marketed as Halal at the time of sampling. Samples were anonymized for analysis and reporting; information identifying individual manufacturers, brands, retail outlets, or certification organizations was not included in the study.

Genomic DNA was extracted using the GMO Extraction Kit (Life Technologies S.A., Alcobendas, Madrid, Spain). Species-specific detection of porcine mitochondrial DNA was performed using the RapidFinder™ Pork ID Kit (Life Technologies S.A., Alcobendas, Madrid, Spain). Real-time PCR amplification and fluorescence detection were performed using a StepOnePlus™ Real-Time PCR System (Applied Biosystems, Foster City, CA, USA).

### 4.2. Genomic DNA Extraction

Genomic DNA was isolated from homogenized food samples using the GMO Extraction Kit (Life Technologies S.A., Alcobendas, Madrid, Spain) according to the manufacturer’s protocol. The resulting DNA extracts were used for subsequent species-specific real-time PCR analysis.

### 4.3. Species-Specific Real-Time PCR Detection of Porcine Mitochondrial DNA

Species-specific detection of porcine (*Sus scrofa*) mitochondrial DNA was performed using the commercial RapidFinder™ Pork ID Kit (Life Technologies S.A., Alcobendas, Madrid, Spain) [14] with a StepOnePlus™ Real-Time PCR System (Applied Biosystems, Foster City, CA, USA). The total reaction volume was 25 µL, comprising 12.5 µL of Total Master Mix, 7.5 µL of Pig Master Mix containing the species-specific assay components, and 5 µL of template DNA at a concentration of approximately 10 ng/µL.

A positive control, a negative extraction control, and a no-template control containing nuclease-free water in place of template DNA were included in the analytical procedure to verify assay performance and monitor potential contamination. PCR amplification performance was additionally monitored using the assay’s internal positive control.

Real-time PCR amplification consisted of an initial denaturation step at 95 °C for 10 min, followed by 36 cycles of denaturation at 95 °C for 15 s and annealing/extension at 60 °C for 60 s. Fluorescence signals were recorded in real time using the StepOnePlus™ system. The qualitative detected/not-detected classification was based on the resulting real-time PCR amplification profiles and the corresponding assay controls.

### 4.4. Experimental Animals and Ethical Approval

Six male BALB/cCrSlc mice obtained from Japan SLC, Inc. (Hamamatsu, Shizuoka, Japan) were used for the controlled spectroscopic experiment. A total of 12 hind-thigh tissue samples were obtained. Each sample consisted of muscle with the femur and tibia retained and the skin removed.

All procedures involving experimental animals were conducted in accordance with applicable institutional guidelines for the care and use of laboratory animals. The experimental protocol was reviewed and approved by the Local Ethics Committee of Asfendiyarov Kazakh National Medical University, Kazakhstan (Protocol No. 20 (156), dated 22 December 2024).

### 4.5. Preparation of Blood-Retained and Blood-Drained Tissue Samples

The experimental animals were assigned to two conditions designed to obtain blood-retained and blood-drained muscle tissues.

For the blood-retained condition, mice were euthanized by an overdose of isoflurane (Mylan Seiyaku Ltd., Tokyo, Japan) without experimental blood removal.

For the blood-drained condition, mice were anesthetized using a SurgiVet inhalation anesthesia system (Smiths Medical PM, Inc., Waukesha, WI, USA) with isoflurane (Mylan Seiyaku Ltd., Tokyo, Japan) at a concentration of 1.0–2.5%. While the animals remained under anesthesia, blood was withdrawn from the caudal vena cava using a syringe equipped with a 22-gauge needle. Up to approximately 1 mL of blood was collected from each animal. The internal jugular veins were subsequently transected to facilitate additional blood removal.

Following the experimental procedures, the hind thighs were dissected. The skin was removed, whereas the femur and tibia remained attached to the muscle tissue. Samples were positioned with the external surface of the hind thigh facing the measurement interface and were subsequently subjected to FT-NIR spectroscopic analysis.

### 4.6. FT-NIR Spectral Acquisition

FT-NIR spectra were acquired using a Vector 22/N FT-NIR spectrometer (Bruker Optik GmbH, Ettlingen, Germany). Spectral measurements were performed over the wavenumber range of 10,000–4000 cm^−1^ at a spectral resolution of 4 cm^−1^. For each measurement, 128 scans were co-added to improve the signal-to-noise ratio.

Spectral data were acquired using OPUS software (version 3.1; Bruker Optik GmbH, Ettlingen, Germany). The resulting absorbance spectra were exported for subsequent chemometric processing and analysis using The Unscrambler software (version 10.1; CAMO AS, Trondheim, Norway).

### 4.7. Spectral Preprocessing and Principal Component Analysis

Prior to multivariate analysis, the raw FT-NIR absorbance spectra were preprocessed using standard normal variate (SNV) transformation to reduce spectral variability associated with light scattering and differences in sample presentation [10,12]. Derivative preprocessing was not applied in the present exploratory analysis; the preprocessing strategy was limited to SNV prior to unsupervised PCA.

Principal component analysis (PCA) was subsequently applied as an unsupervised multivariate method to reduce the dimensionality of the spectral dataset and explore systematic variation between blood-retained and blood-drained tissue samples [11]. PCA score plots were used to visualize the distribution and clustering of samples by experimental condition. The corresponding loading profiles were examined to identify the spectral regions contributing most strongly to the observed separation between the two groups.

## 5. Conclusions

This study demonstrated the complementary potential of molecular and spectroscopic approaches for meat authentication. Species-specific real-time PCR detected porcine mitochondrial DNA in 64 of 85 (75.3%) commercially available meat and meat-containing products analyzed in the present sample set, supporting the use of molecular analysis for detecting species-specific animal-derived genetic material in complex food matrices. Importantly, detection of porcine DNA establishes the presence of the target genetic material but does not, by itself, distinguish among intentional species substitution, ingredient-related sources, or unintended cross-contact during processing.

In the controlled experimental component, FT-NIR spectroscopy combined with SNV preprocessing and PCA revealed systematic multivariate differences between blood-retained and blood-drained murine muscle tissues. These differences were consistent with changes in the overall tissue composition associated with the blood-retained and blood-drained experimental conditions. Water-related variation may contribute substantially to the observed spectral separation; however, because tissue moisture, residual blood, and hemoglobin were not independently quantified, the relative contributions of these factors cannot be determined from the present data. Accordingly, the observed separation should not be attributed to a specific biochemical constituent.

The two analytical approaches provide complementary information: real-time PCR offers species-specific molecular identification, whereas FT-NIR spectroscopy provides rapid and non-destructive characterization of broader compositional differences. Together, these approaches provide a basis for a potential framework for multidimensional meat authentication and food analysis.

Nevertheless, the spectroscopic component of this study should be considered a proof of concept rather than a validated diagnostic method for determining slaughter status. Further studies using larger datasets, commercially relevant livestock species, independent measurements of tissue moisture, residual blood, and hemoglobin, and externally validated chemometric classification models are required to establish analytical performance and practical applicability. Future integration of validated molecular confirmation with rapid spectroscopic screening may provide an effective strategy for food authenticity assessment and food safety monitoring.

## Figures and Tables

**Table 1:** Illustrative examples of qualitative real-time polymerase chain reaction (real-time PCR) results from selected product categories within the analyzed sample set.

Product Type	Halal-Labeled	Porcine DNA
Beef sausage	Yes	Detected
Chicken sausage	Yes	Detected
Mixed minced meat	Yes	Detected
Beef cutlets	Yes	Detected
Lamb dumplings	Yes	Detected
Beef minced meat	Yes	Detected
Seasoning mixture	Yes	Detected
Raw beef	Yes	Not detected

## Data Availability

The data supporting the findings of this study are available from the corresponding author upon reasonable request. The data are not currently deposited in a publicly accessible repository.
